# Short–term outcomes of heavyweight versus mediumweight synthetic mesh in a retrospective cohort of clean–contaminated and contaminated retromuscular ventral hernia repairs

**DOI:** 10.1007/s00464-024-10946-0

**Published:** 2024-06-11

**Authors:** Ryan C. Ellis, Sara M. Maskal, Nir Messer, Benjamin T. Miller, Clayton C. Petro, Ajita S. Prabhu, Michael J. Rosen, Xinyan Zheng, Lucas R. A. Beffa

**Affiliations:** 1 Department of Surgery, Cleveland Clinic Center for Abdominal Core Health, Cleveland Clinic Foundation, 9500 Euclid Avenue, Cleveland, OH 44195 USA; 2 Department of Population Health Sciences, Weill Cornell College of Medicine, New York, NY USA

**Keywords:** Hernia, Heavyweight, Contamination, Permanent mesh

## Abstract

**Background:**

Mediumweight (40–60 g/m^2^) polypropylene (MWPP) mesh has been shown to be safe and effective in CDC class II–III retromuscular ventral hernia repairs (RMVHR). However, MWPP has the potential to fracture, and it is possible that heavyweight (> 75 g/m^2^) polypropylene mesh has similar outcomes in this context. However, there is limited data on HWPP mesh performance in clean-contaminated and contaminated scenarios. We aimed to compare HWPP to MWPP mesh in CDC class II–III wounds during open RMVHR.

**Methods:**

The Abdominal Core Health Quality Collaborative database was retrospectively queried for a cohort of patients who underwent open RMVHR with MWPP or HWPP mesh placed in CDC class II/III wounds from 2012 to 2023. Mesh types were compared using a 3:1 propensity score-matched analysis. Covariates for matching included CDC classification, BMI, diabetes, smoking within 1 year, hernia, and mesh width. Primary outcome of interest included wound complications. Secondary outcomes included reoperations and readmissions at 30 days.

**Results:**

A total of 1496 patients received MWPP or HWPP (1378 vs. 118, respectively) in contaminated RMVHR. After propensity score matching, 351 patients remained in the mediumweight and 117 in the heavyweight mesh group. There were no significant differences in surgical site infection (SSI) rates (13.4% vs. 14.5%, *p* = 0.877), including deep SSIs (0.3% vs. 0%, *p* = 1), surgical site occurrence rates (17.9% vs. 22.2%, *p* = 0.377), surgical site occurrence requiring procedural intervention (16% vs. 17.9%, *p* = 0.719), mesh removal (0.3% vs. 0%, *p* = 1), reoperations (4.6% vs. 2.6%, *p* = 0.428), or readmissions (12.3% vs. 9.4%, *p* = 0.504) at 30 days.

**Conclusion:**

HWPP mesh was not associated with increased wound morbidity, mesh excisions, reoperations, or readmissions in the early postoperative period compared with MWPP mesh in open RMVHR for CDC II/III cases. Longer follow-up will be necessary to determine if HWPP mesh may be a suitable alternative to MWPP mesh in contaminated scenarios.

Mesh selection for use in contaminated ventral hernia repairs can be a difficult decision and the use of permanent synthetic mesh in this setting remains controversial despite several studies showing safety [1–5]. Surgeons often consider using a biologic mesh in these challenging clinical scenarios because of the contaminated nature of these cases. The use of biologic mesh versus synthetic mesh was compared in a recent randomized trial by Rosen et al., which showed increased recurrence rates with biologic mesh, increased cost, and no differences in wound complications between biologic and mediumweight polypropylene (MWPP) (40–60 g/m^2^) synthetic retromuscular mesh in contaminated cases [6]. The safety, efficacy, and overall success of synthetic mesh in contaminated settings have been attributed to the macroporous and weight properties of MWPP.

Recently, concerns over the durability of MWPP have been raised due to reports of central mesh fracture, particularly after bridged hernia repairs [7]. Heavyweight (mesh density > 75 g/m^2^) polypropylene mesh (HWPP) has been evaluated in clean cases and may represent a suitable alternative to MWPP demonstrated in a large clinical trial; however, all of these cases were clean and therefore, the performance of HWPP has not been critically evaluated in clean-contaminated and contaminated cases [8].

When considering the utilization of HWPP in these complex scenarios, an additional limitation is the size of the mesh produced. While a 50 × 50 cm MWPP is currently on the market, only a 30 × 30 cm HWPP mesh was commercially available and approved for use in the USA at the time of this analysis. In rare circumstances when a larger piece of HWPP mesh is required for adequate mesh overlap, several pieces of 30 × 30 cm mesh can be sewed together into a paneled configuration with permanent suture to create a larger prosthetic (Fig. 1). So, an additional concern with an analysis of HWPP in these complicated cases is the confounding nature of paneled mesh with additional suture lines and knots which may influence outcomes.

**Fig. 1:**
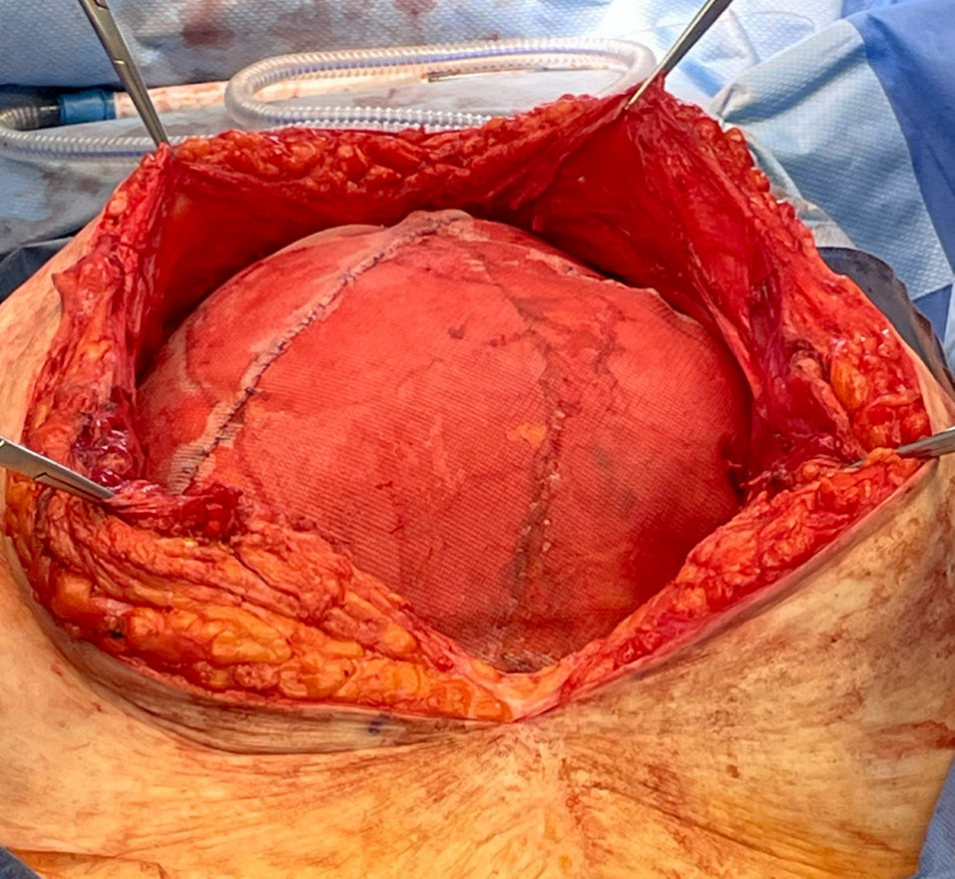
An example of paneled HWPP mesh with several sheets sewn together using permanent suture

With this lack of clinical evaluation of HWPP in non-clean cases, the question remains of how HWPP performs in contaminated scenarios compared to MWPP. We aimed to investigate and compare short-term outcomes of HWPP (both non-paneled and paneled configurations) to MWPP mesh in contaminated retromuscular ventral hernia repair.

## Methods

Following approval from the institutional review board (IRB) at the Cleveland Clinic Foundation (IRB 23–346), a cohort of adult patients who received either MWPP or HWPP mesh during retromuscular ventral hernia repairs in center for disease control and prevention (CDC) class II and III cases between January 2012 and June 2023 were identified retrospectively from the abdominal core health quality collaborative (ACHQC). The research protocol and IRB approval were completed prior to obtaining the data from the ACHQC. This trial was registered retrospectively on ClinicalTrials.gov. CDC class I and IV cases were excluded. The ACHQC is a prospectively collected, surgeon-entered registry to record patient characteristics, operative details, and outcomes [9].

We collected data for 30 day complications. Primary outcomes included wound complications, including surgical site infections (SSIs), surgical site occurrences (SSOs), and surgical site occurrences requiring procedural intervention (SSOPIs). Secondary outcomes included hernia recurrences, reoperations, and readmissions. A 3:1 propensity score-matched (PSM) analysis was performed comparing short-term outcomes of MWPP and HWPP mesh. Covariates used in the PSM analysis included wound classification, BMI, diabetes, smoking within 1 year, hernia width, and mesh width.

An additional subgroup analysis was performed comparing repairs requiring single sheets of HWPP (identified as mesh width ≤ 30 cm) with those requiring suturing together (or paneled) sheets of HWPP. Paneled meshes were identified within the database as HWPP mesh width > 30 cm. A 1:1 PSM was performed for this sub-analysis, with covariates consisting of CDC class, BMI, diabetes, smoking within 1 year, and hernia width. The same outcomes were compared as previously stated.

Both PSM analyses were performed using nearest neighbor matching on the logit of the propensity score with a caliper width of 0.2 of the standard deviations of the logit of the propensity score. Categorical variables were summarized as frequencies (*n*) and percentages (%) and compared using Pearson’s Chi-squared test or Fisher’s exact test, as appropriate. Continuous variables were summarized as median with interquartile range (IQR, 25th and 75th percentile) and compared using Wilcoxon rank sum test. Statistical analyses were performed using R software (version 4.2.2; R Core Team, R Foundation for Statistical Computing, Vienna, Austria).

## Results

**Fig. 2:**
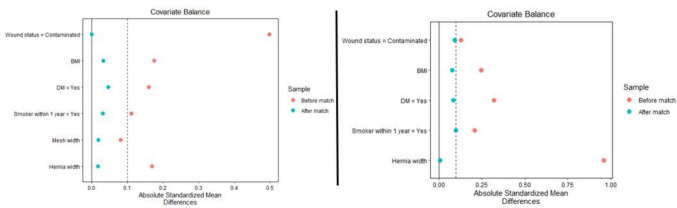
Distributions before and after propensity score matching for the MWPP versus HWPP (left) and single-sheet HWPP versus paneled HWPP (right)

A total of 1496 patients were identified within the ACHQC database as having received MWPP or HWPP (1378 vs. 118, respectively) in contaminated (CDC class II or III) retromuscular ventral hernia repairs. After propensity score matching, a total of 468 patients remained with 351 in the MWPP group and 117 in the HWPP group (Fig. 2). Baseline characteristics were similar between the two groups (Table 1).

**Table 1:** Baseline variables and operative characteristics, after propensity score matching

	MWPP (*n* = 351)	HWPP (*n* = 117)
Age, median [IQR], years	60 [49, 68]	59 [51, 66]
Gender		
Female	192 (55%)	68 (58%)
Male	149 (45%)	49 (42%)
BMI (capped 15–60)	33 [29, 37]	33 [29, 37]
Diabetes Mellitus	112 (32%)	35 (30%)
Anti-coagulation medications	29 (8.3%)	11 (9.4%)
ASA class		
1	3 (0.9%)	2 (1.7%)
2	65 (19%)	29 (25%)
3	270 (77%)	82 (70%)
4	13 (3.7%)	4 (3.4%)
History of abdominal wall surgical site infection	100 (28%)	28 (24%)
Currently active infection	13 (3.7%)	3 (2.6%)
History of inflammatory bowel disease	52 (15%)	11 (9.4%)
History of component separation	26 (7.4%)	14 (12%)
Immunosuppression	26 (7.4%)	7 (6%)
Smoker within one year	59 (17%)	21 (18%)
Elective case	342 (97%)	113 (97%)
Wound status		
Clean	0	0
Clean-contaminated	282 (80%)	94 (80%)
Contaminated	69 (20%)	23 (20%)
Dirty/infected	0	0
Operative time, min		
0–59	0	0
60–119	5 (1.4%)	4 (4.3%)
120–179	56 (16%)	27 (23%)
180–239	82 (23%)	31 (26%)
240 +	208 (59%)	54 (46%)
Intra-op complications	61 (17%)	15 (13%)
Stoma present	141 (40%)	19 (16%)
Subcutaneous flaps raised	45 (13%)	27 (23%)
Mesh width	30.0 [24.0, 30.5]	30.0 [25.0, 38.5]
Hernia dimensions, cm		
Hernia width	14.0 [10, 18]	14 [10, 19]
Hernia length	22 [15, 26]	23 [16, 25]

*ASA* American Society of Anesthesiologists

Data reported as median [IQR] or *n* (%) where appropriate

Short-term wound morbidity outcomes between MWPP and HWPP mesh were similar. Table 2 shows the breakdown of all 30 day complications. There were no significant differences between MWPP and HWPP groups regarding surgical site infection rates (13.4% vs. 14.5%, *p* = 0.877), surgical site occurrence rates (17.9% vs. 22.2%, *p* = 0.377), SSOPI rates (16% vs. 17.9%, *p* = 0.719), and mesh removal (0.3% vs. 0%, *p* = 1), respectively.

**Table 2:** 30 day outcomes

	MWPP (*n* = 351)	HWPP (*n* = 117)	*p*-value
Recurrence	0	0	–
Surgical site infection (SSI)	47 (13.4%)	17 (14.5%)	0.877
Superficial	29 (8.3%)	8 (6.9%)	0.778
Deep incisional	15 (4.3%)	9 (7.8%)	0.221
Organ space	5 (1.4%)	0	0.339
Surgical site occurrence (SSO)	63 (17.9%)	26 (22.2%)	0.377
Wound cellulitis	11 (3.1%)	4 (3.4%)	1
Non-healing wound	4 (1.1%)	2 (1.7%)	0.643
Fascial disruption	3 (0.9%)	2 (1.7%)	0.603
Skin or soft-tissue ischemia	1 (0.3%)	1 (0.9%)	0.439
Serous drainage	12 (3.4%)	6 (5.1%)	0.411
Purulent drainage	6 (1.7%)	5 (4.3%)	0.153
Seroma	20 (5.7%)	6 (5.1%)	0.995
Hematoma	9 (2.6%)	4 (3.4%)	0.745
Exposed mesh	2 (0.6%)	1 (0.9%)	1
Enterocutaneous fistula	1 (0.3%)	0	1
Mesh infection	1 (0.3%)	0	1
SSI/SSO procedural interventions	56 (16%)	21 (17.9%)	0.719
Oral antibiotics	16 (4.6%)	11 (9.4%)	0.086
IV antibiotics	7 (2%)	5 (4.3%)	0.185
Percutaneous drainage	6 (1.7%)	3 (2.6%)	0.697
Wound opening	17 (4.8%)	5 (4.3%)	1
Wound debridement	5 (1.4%)	3 (2.6%)	0.419
Suture excision	0	0	–
Partial mesh removal	0	0	–
Complete mesh removal	1 (0.3%)	0	1
Reoperation	16 (4.6%)	3 (2.6%)	0.428
Readmission	43 (12.3%)	11 (9.4%)	0.504
Respiratory insufficiency requiring reintubation	9 (2.6%)	2 (1.7%)	0.739
Bleeding requiring transfusion	6 (1.7%)	7 (6%)	0.023
Bowel obstruction	3 (0.9%)	1 (0.9%)	1
Ileus	41 (11.8%)	19 (16.4%)	0.273
Deep venous thrombosis (DVT)	6 (1.7%)	1 (0.9%)	0.686
Pulmonary embolism	4 (1.1%)	1 (0.9%)	1
Stroke	0	0	-
Septic shock	0	0	-
Myocardial infarction	0	1 (0.9%)	0.25

Data reported as *n* (%)

Among the remaining 30 day outcomes, bleeding events requiring transfusion occurred significantly more in the HWPP group (1.7% vs. 6%, *p* = 0.023). All other 30 day outcomes remained not significant between the MWPP and HWPP groups, including recurrence (0 vs. 0), reoperations (4.6% vs. 2.6%, *p* = 0.428), or readmissions (12.3% vs. 9.4%, *p* = 0.504), respectively.

We then performed an analysis of single sheet HWPP compared to a paneled HWPP repair (Fig. 2). After 1:1 PSM, 27 patients remained in each group: single sheet vs. paneled. The breakdown of baseline characteristics and operative details are found in Table 3 and 30 day outcomes in Table 4. Among the baseline characteristics, the single-sheet HWPP versus paneled HWPP were well matched. Like the previous analysis, all cases consisted of CDC class 2 and 3-contaminated wounds. At 30 days, there were no significant differences between single-sheet or paneled mesh groups with similar SSI rates (11.1% vs. 11.1%, *p* = 1), SSO rates (18.5% vs. 29.6%, *p* = 0.524), and SSOPI rates (11.1% vs. 22.2%, *p* = 0.467), respectively.

**Table 3:** Baseline characteristics and operative details of the single sheet and paneled HWPP mesh, after propensity score matching

	Single-Sheet HWPP (n = 27)	Paneled HWPP (n = 27)
BMI, median [IQR]	33.1 [30.2, 36.8]	31.6 [27.6, 36.2]
Diabetes	7 (25.9%)	8 (29.6%)
Smoker within one year	5 (18.5%)	4 (14.8%)
Wound status		
Clean	0	0
Clean-contaminated	22 (81.5%)	21 (77.8%)
Contaminated	5 (18.5%)	6 (22.2%)
Dirty/infected	0	0
Hernia width, median [IQR], cm	15 [12, 19]	15 [11.5, 20]
Fascial closure was achieved	22 (81%)	20 (74%)

Data reported as median [IQR] or *n* (%) where appropriate

**Table 4:** Short-term outcomes of surgeries requiring single sheet vs. paneled mesh

	Single-sheet HWPP (*n* = 27)	Paneled HWPP (*n* = 27)	*p*-value
Recurrence	0	0	–
Surgical site infection (SSI)	3 (11.1%)	3 (11.1%)	1
Superficial	1 (3.7%)	1 (3.7%)	1
Deep incisional	3 (11.1%)	2 (7.4%)	1
Organ space	0	0	–
Surgical site occurrence (SSO)	5 (18.5%)	8 (29.6%)	0.524
Wound cellulitis	2 (7.4%)	1 (3.7%)	1
Non-healing wound	1 (3.7%)	0	1
Fascial disruption	0	0	–
Skin or soft-tissue ischemia	0	0	–
Serous drainage	0	4 (14.8%)	0.111
Purulent drainage	1 (3.7%)	1 (3.7%)	1
Seroma	1 (3.7%)	2 (7.4%)	1
Hematoma	0	0	–
Exposed mesh	0	0	–
Enterocutaneous fistula	0	0	–
SSI/SSO procedural interventions	3 (11.1%)	6 (22.2%)	0.467
Oral antibiotics	3 (11.1%)	3 (11.1%)	1
IV antibiotics	3 (11.1%)	1 (3.7%)	0.61
Percutaneous drainage	1 (3.7%)	1 (3.7%)	1
Wound opening	0	3 (11.1%)	0.236
Wound debridement	0	2 (7.4%)	0.491
Suture excision	0	0	–
Partial mesh removal	0	0	–
Complete mesh removal	0	0	–
Reoperation	0	1 (3.7%)	1
Readmission	2 (7.4%)	1 (3.7%)	1
Respiratory insufficiency requiring reintubation	0	1 (3.7%)	1
Bleeding requiring transfusion	2 (7.4%)	1 (3.7%)	1
Bowel obstruction	0	0	–
Ileus	7 (25.9%)	1 (3.7%)	0.05
Deep venous thrombosis (DVT)	0	0	–
Pulmonary embolism	0	0	–
Stroke	0	0	–
Septic shock	0	0	–
Myocardial infarction	0	0	–

Data reported as *n* (%)

## Discussion

In this retrospective registry-based PSM analysis, MWPP and HWPP were found to have similar short-term outcomes when used in contaminated open retromuscular ventral hernia repairs. These similarities were also found between single sheet and paneled sheets of HWPP mesh.

The successful use of polypropylene mesh in contaminated (CDC II/III) retromuscular hernia repairs has been consistently reported over the past decade, as the advent of a transversus abdominis release has allowed for wide adoption of retromuscular techniques for complex ventral hernias [10, 11]. The largest series of 402 patients by Warren et al. reported a 2.4% rate of mesh excision and 0 enterocutaneous fistula with 21 month median follow-up. Likewise, our recent randomized trial comparing MWPP to biologic mesh found that the synthetic mesh had similar rates of wound morbidity and was more durable at 2 years. However, reports of the effective use of polypropylene in this setting have exclusively focused on lightweight (density < 40 g/m^2^) and mediumweight (density 40–60 g/m^2^) polypropylene and its success has been attributed to its density and macroporosity. Theoretically, lighter weight/larger pored materials allow for increased flexibility, tissue ingrowth, and bacterial clearance [7, 12, 13]. However, we have considered the possibility that polypropylene mesh weight and porosity are not as critical to its resilience as is often regarded and that HWPP (density > 75 g/m^2^) may perform just as well in contaminated cases of elective open retromuscular hernia repair. Our data in the context of elective retromuscular repairs is consistent with the initial studies of HWPP almost 70 years ago that highlighted the material’s impressive resistance to microorganisms [14]. More contemporary studies support antimicrobial properties of polypropylene mesh with resistance to MRSA adherence [15, 16].

Apprehension to using synthetic mesh in contaminated settings pre-dated modern retromuscular techniques and ironically originated with the use of HWPP (Marlex®) mesh in complicated cases of intra-abdominal catastrophe. Marlex® 50 (Bard-Davol, Cranston, RI), a heavyweight mesh employed in the use of hernia repairs, eventually led to the adoption of Marlex® by the US military for use in closing contaminated abdomens with large defects and other highly contaminated scenarios [17–19]. Subsequently, Voyles et al. reported their 5 year experience using Marlex® intraperitoneally in 31 heavily contaminated ventral hernia repairs, 25 consisting of necrotizing soft-tissue infections (many with associated intra-abdominal sepsis) and found that early success was curtailed with late development of enterocutaneous fistula development and wound complications requiring mesh excision warranted second thought to the use of this permanent material [20]. Other studies performed in the 1980s made similar conclusions to Voyles et al. with significant wound morbidity issues at long-term follow-up time points [21]. When considering our contemporary use of HWPP in contaminated scenarios, the importance of distinguishing that these are elective cases with a prosthetic in a well-vascularized retromuscular plane cannot be underscored enough. The absence of mesh excisions or enterocutaneous fistulas in our HWPP cohort begin to validate that this context is separate and distinct from those remote cases but also emphasizes the importance of long-term follow-up.

The use of mediumweight monofilament polypropylene seems to be effective in the majority of context during the perioperative period; however, our group became concerned due to a recent analysis that demonstrated a 4.2% fracture rate of MWPP at 1 year following open retromuscular ventral hernia repair, a rate that increased to 30% in bridged scenarios [22]. In an era when retromuscular repairs are becoming ubiquitous, we are concerned that rates of mesh fracture may increase, particularly with longer follow-up and leave a large contingency of patients needing another repair after the retromuscular space has been utilized. Furthermore, for contaminated (CDC II/III) scenarios when concomitant abdominal wall reconstruction is being entertained, this rate of mesh fracture adds another layer of complexity when considering delaying definitive hernia repair and staging reconstruction. As such, an additional impetus for this analysis was to investigate whether HWPP could be utilized in these concomitant cases to allow for similar durability and the avoidance of another operation.

Today, we are less hesitant to utilize HWPP in CDC II/III fields. A residual limitation remains scenarios where wide mesh overlap is needed and anterior fascial coverage is not possible, leaving a bridged scenario. In this scenario, where MWPP is most vulnerable to mesh fracture, we either utilize a 50 × 50 cm MWPP or paneled HWPP. This drove a sub-analysis of the single sheet vs. paneled HWPP mesh, which did not show any differences in outcomes at 30 days. As mesh industry continues to advocate for absorbable meshes at astronomical cost, perhaps a simpler approach would be to create larger sheets of an already well-studied product. At the time of this article, Surgimesh® (BG Medical, Deer Park, IL), is made in larger HWPP sheets; however, the outcomes of this multifilament alternative are currently unknown.

This study has several limitations to address. The overall number of patients included in analysis is small and may not accurately reflect the true incidence of complications in this population. Many surgeons feel that 30 day outcomes are not long enough, even for short-term wound complications. Certainly, longer-term follow-up is needed to truly comment on the safe use of HWPP mesh in contaminated settings. While propensity score matching helps to equalize comparator groups, the retrospective nature of this data introduces the potential for selection bias. The ACHQC, while nationwide, may not represent common practice as many surgeons that participated in the ACHQC are hernia experts with high level of interest in hernia surgery, thus these results may not be generalizable.

## Conclusion

This study demonstrates similar short-term outcomes between MWPP and HWPP mesh in clean-contaminated and contaminated retromuscular hernia repairs.
